# Acute Responses to High-Intensity Back Squats with Bilateral Blood Flow Restriction

**DOI:** 10.3390/ijerph20043555

**Published:** 2023-02-17

**Authors:** Bjoern Hornikel, Keith S. Saffold, Michael R. Esco, Jacob A. Mota, Michael V. Fedewa, Stefanie A. Wind, Tiffany L. Adams, Lee J. Winchester

**Affiliations:** 1 Department of Epidemiology, The University of Alabama at Birmingham, Birmingham, AL 35294, USA; 2 Department of Kinesiology, The University of Alabama, Tuscaloosa, AL 35487, USA; 3 Department of Kinesiology and Sport Management, Texas Tech University, Lubbock, TX 79409, USA; 4 Department of Educational Studies in Psychology, Research Methodology, and Counseling, The University of Alabama, Tuscaloosa, AL 35487, USA

**Keywords:** occlusion training, resistance exercise, blood flow restriction, metabolic stress, fatigue

## Abstract

This study examined the acute effects of high-intensity resistance exercise with blood flow restriction (BFR) on performance and fatigue, metabolic stress, and markers of inflammation (interleukin-6 (IL-6)), muscle damage (myoglobin), angiogenesis (vascular endothelial growth factor (VEGF)). Thirteen resistance-trained participants (four female, 24.8 ± 4.7 years) performed four sets of barbell back-squats (75% 1RM) to failure under two conditions: blood flow restriction (BFR, bilateral 80% occlusion pressure) and control (CTRL). Completed repetitions and pre–post-exercise changes in maximal voluntary isometric contractions, countermovement jump, barbell mean propulsive velocity, and surface electromyography were recorded. Pre–post blood lactate (BLa) and venous blood samples for analysis of IL-6, myoglobin, and VEGF were collected. Ratings of perceived exertion (RPE) and pain were recorded for each set. Fewer repetitions were performed during BFR (25.5 ± 9.6 reps) compared to CTRL (43.4 ± 14.2 reps, *p* < 0.001), with greater repetitions performed during sets 1, 2, and 4 (*p* < 0.05) in CTRL. Although RPE between conditions was similar across all sets (*p* > 0.05), pain was greater in BFR across all sets (*p* < 0.05). Post-exercise fatigue was comparable between conditions. BLa was significantly greater in CTRL compared to BFR at two minutes (*p* = 0.001) but not four minutes post-exercise (*p* = 0.063). IL-6 was significantly elevated following BFR (*p* = 0.011). Comparable increases in myoglobin (*p* > 0.05) and no changes in VEGF were observed (*p* > 0.05). BFR increases the rate of muscular fatigue during high-intensity resistance exercise and acutely enhances IL-6 response, with significantly less total work performed, but increases pain perception, limiting implementation.

## 1. Introduction

Muscle hypertrophy, or the enlargement of individual muscle fibers, is an outcome of acute neural, hormonal, and muscular responses to resistance training, which leads to long-term adaptations in muscle hypertrophy and strength [1,2]. Contemporary muscle hypertrophy recommendations focus on high-intensity resistance training (HI-RT) using a workload of >65% of 1-repetition-max (1RM) to maximize hypertrophic adaptations [3]. Previous research has demonstrated that adaptations of muscle hypertrophy in response to HI-RT are primarily related to mechanical tension and metabolic stress occurring as a result of the load placed on the muscle fiber during exercise [4]. The high load associated with HI-RT aids in activating both of these mechanisms, leading to an increase in protein synthesis [5].

More recently, blood flow restriction (BFR) resistance exercise has demonstrated its utility as an alternative method for inducing muscle hypertrophy. BFR exercise involves the use of a tourniquet, pneumatic cuff, or even elastic wraps to occlude distal blood flow in a limb. External pressure to the limb, by application and pressurization of the tourniquet, reduces arterial blood supply and venous return, with higher pressures restricting blood flow to a greater extent. This leads to an ischemic and hypoxic muscular environment causing high levels of metabolic stress. BFR in combination with low-intensity resistance training (20–30% 1RM) has been shown to produce greater muscle hypertrophy compared to low-intensity resistance training without BFR [6,7] and similar hypertrophic increases compared to traditional HI-RT [8,9].

Whereas hypertrophic response from HI-RT is associated with high mechanical tension and possibly muscle damage [10], low-intensity BFR (LI-BFR) exercise induces hypertrophy through alternative mechanisms [11]. Indeed, during BFR exercise, metabolic stress is believed to be the primary mechanism for hypertrophy [12]. Restricted blood flow during exercise creates a hypoxic environment, leading to increased rates of ATP hydrolysis, phosphocreatine (PCr) depletion, decreased pH, and increased lactate response [13]. LI-BFR produces greater metabolic stress compared to LI-RT [12,14], and enhances metabolic stress to the level of HI-RT [14].

Both HI-RT and LI-BFR are well-researched, effective modalities for promoting muscle hypertrophy. However, less is known about the potential utility of high-intensity BFR exercise (HI-BFR) to further enhance the hypertrophic capacity by maximizing mechanical tension and enhancing metabolic stress through restricted venous blood flow.

Previous literature has demonstrated the occurrence of enhanced recruitment of higher-threshold motor units during LI-BFR [15,16,17]. Type 2 fibers, which have greater prevalence in high-threshold motor units, have an ability to achieve 3 to 4 times greater protein synthesis through p70S6k expression, a downstream target of mammalian target of rapamycin (mTOR), compared to T1 fibers, indicating a greater potential for hypertrophy [18]. Reduced oxygen supply and accumulation of metabolites during LI_BFR stimulates group 3 and 4 afferents, which inhibit slow-twitch alpha motoneurons, leading to an increase in fast-twitch fiber recruitment to maintain force output [18,19]. However, when comparing electromyography (EMG) for muscle activation, EMG amplitude during LI-BFR is lower than that experienced during HI-RT [19,20]. Therefore, the mechanical tension produced by HI-RT may play an additional role alongside metabolic stress through more potent T2 fiber recruitment.

Additionally, high resistance loads increase the mechanical tension and subsequent muscle damage associated with RT, which are key anabolic signals for activating the mitogen activated protein kinase (MAPK) protein synthesis pathway, leading to increased protein synthesis and hypertrophy [19]. Most recently, Winchester et al. demonstrated that unilateral BFR during HI back-squats (75% 1RM) did not increase markers of muscle damage (IL-6 and myoglobin) over non-BFR HI back-squats [20]. However, muscular fatigue was induced more rapidly with BFR, suggesting that other mechanisms of hypertrophy are plausible.

The limited studies examining HI-BFR have demonstrated its utility for increased blood lactate concentrations [21], enhanced rate of fatigue [20,22], and reduced post-exercise muscle activation [21,23]. The purpose of this study was to examine the feasibility and acute physiological and perceptual effects of bilateral HI-BFR barbell back-squats on performance and fatigue, metabolic stress, and plasma markers of muscle damage (myoglobin), inflammation (IL-6), angiogenesis (VEGF). This study will add to the limited HI-BFR knowledge and shed light on the practical utility and efficacy of HI-BFR among resistance-trained individuals. The authors hypothesized that the addition of BFR during HI-RT would acutely increase fatigue, during and post-exercise, increase metabolic stress and angiogenesis, without an increase in markers of muscle damage or inflammation compared to traditional HI-RT following the exercise protocol.

## 2. Materials and Methods

### 2.1. Study Design Overview

Participants attended the laboratory on three separate occasions over a three-week period in a counterbalanced, cross-over design. Each individual participant’s visits were separated by one week, and the two experimental trials were performed at approximately the same time (±1 h) of day. Participants were asked to refrain from any moderate-to-vigorous physical activity for 72 h prior to each laboratory visit during the study [24]. To mitigate dietary influence on performance, participants were asked to eat a similar diet prior to their laboratory visits throughout the study.

The first visit served to provide participants with an explanation of study procedures and familiarization with study equipment. This was followed by assessment of baseline participant characteristics (height, body mass, and resting blood pressure (BP)) and familiarization with perceptual measures (visual analog scale-delayed-onset muscle soreness (VAS-DOMS) and perceptual recovery status (PRS)). Lastly, the barbell back-squat 1RM testing was completed.

During the second and third visits, participants completed two experimental trials in a counterbalanced order. Upon arrival at the laboratory, participants completed VAS-DOMS and PRS measures followed by measures of resting BP and body mass. Next, baseline biomarkers (finger prick blood lactate and venous blood samples) were collected prior to the warm-up. Participants completed a 5-min warm-up on a cycle ergometer, followed by a self-selected dynamic warm-up (i.e., arm circles, lunges, walking hamstring stretch, bodyweight squats). Participants then completed a series of non-fatiguing performance measures: maximal voluntary isometric contraction (MVIC), countermovement jump (CMJ), and mean propulsive velocity (MPV). Next, participants completed one of two resistance exercise protocols: 1. traditional high-intensity resistance exercise (CTRL: 75% 1RM, 4 sets to failure); and 2. high-intensity resistance exercise with BFR (BFR: 75% 1RM, 4 sets to failure, 80% LOP), followed by post-exercise biomarker and performance measurements.

### 2.2. Participants

Thirteen (4 female, 24.8 ± 4.7 yrs, 177.8 ± 11.8 cm, 84.3 ± 16.7 kg) apparently healthy adults were recruited to participate in this study. All participants were non-smokers, classified as advanced resistance-trained (i.e., minimum of 1-year resistance training experience with at least 3 sessions per week) [25], and incorporated barbell back squats routinely in their resistance training program. Participant characteristics and pre-testing 1RMs can be seen in Table 1. An a priori power analysis was performed (G*Power, version 3.1.9.6, Universität Kiel, Kiel, Germany) following the recommendations of Beck [26]. Using repeated-measures, within-factors ANOVA, effect size of 0.50, alpha (α) level of 0.05, desired power (1−β) of 0.80, 2 groups, 2 measurements, and correlation among repeated measures of 0.5 would require a total sample size of 12 participants to detect the anticipated effect. However, to account for potential participant attrition or incomplete blood samples, additional participants were recruited for the study. Participants were excluded if they did not participate in regular resistance training exercise, if they self-reported cardiovascular, metabolic, or pulmonary conditions or signs and symptoms suggestive of these diseases [24], if their resting systolic BP (BP) ≥ 140 mmHg and/or diastolic BP ≥ 90 mmHg [27], or if it became apparent that they were unable to achieve the full range of motion during back-squats required for this study. This study was approved by the University Institutional Review Board. All participants provided written informed consent in accordance with the Declaration of Helsinki.

### 2.3. Procedures

#### 2.3.1. Baseline Characteristics

Standing height was measured to the nearest 0.1 cm using a stadiometer (SECA 67310, SECA©, Chino, CA, USA). Body mass was measured to the nearest 0.1 kg on a digital scale (Tanita BWB-800, Tanita©, Arlington Heights, IL, USA). Percent body fat estimations were measured during the initial familiarization visit via 7-site skinfold assessment (Lange Skinfold Caliper, Beta Technology Inc., Cambridge, MD, USA), with body density calculated using sex-specific Jackson–Pollock equations [28]. Body fat (BF%) was calculated from body density using the Siri equation: %Fat = (4.95/body density − 4.50) × 100 [29]. Resting BP was determined after 5 min of seated rest, BP was measured with the BPM-100 automated BP monitor (BPtru medical devices) three times, 1 min apart in the dominant arm and averaged [27].

#### 2.3.2. Perceptual Measures

To ensure similar expected performance between visits, participants completed a visual analog scale-delayed-onset muscle soreness (VAS-DOMS) and perceptual recovery status (PRS). VAS-DOMS was measured along a 10 cm line with “no pain” on one end, and “unbearable pain” on the other end [30]. Participants were instructed to mark a vertical line along the 10 cm line [30]. DOMS was rated as the measured distance between the marked line and the left end (no pain) of the scale [30]. PRS was assessed using a 0–10 scale, with 2 sets of verbal anchors defining the numerical indicators of both recovery and expected performance [31]. A score of zero indicates that the individual is “very poorly” recovered and may perform poorly whereas a score of 10 would denote that the athlete is fully recovered and will perform optimally [31].

#### 2.3.3. RM Testing

Prior to 1RM testing during the first visit, participants warmed up on a cycle ergometer for 5 min, followed by a self-selected dynamic warm-up (i.e., arm circles, lunges, walking hamstring stretch, bodyweight squats). Following the warm-up, barbell back-squat 1RM was evaluated, following National Strength and Conditioning Association recommended guidelines [32]. Participants were asked to flex their knee and lower their hips to the point that the femur was parallel to the ground followed by full extension. A strength and conditioning specialist observed squat depth and technique during all repetitions. The 1RM was determined by the maximum weight the participant was able to squat while maintaining proper exercise technique.

#### 2.3.4. Resistance Exercise Protocols

The two resistance exercise protocols, BFR and CTRL, consisted of high-intensity barbell back-squats (75% 1RM) with and without blood flow restriction, respectively. During both protocols, participants performed four sets of barbell back-squats at 75% of 1RM with three minutes rest between sets. Each set was performed to failure, with the number of repetitions successfully completed recorded for each set. Prior to the BFR protocol, participants were fitted with inflatable pneumatic cuffs (11.5 cm width) positioned within 4 inches of the inguinal crease on both legs. Next, LOP was determined by the Delfi Personalized Tourniquet System II (PTS II) (Delfi Medical, Vancouver, BC, Canada), with participants in a seated position and each leg measured in turn. During the BFR protocol, bilateral occlusion at 80% LOP was applied to the lower limbs. The cuffs were inflated 30 s before beginning the first set and remained inflated until the completion of the second set, at which point the cuffs were deflated, allowing for approximately 2 min of reperfusion between these sets. The cuffs were re-inflated 30 s prior to the start of set 3 and remained inflated until 2 min after set 4 was completed.

#### 2.3.5. Blood Sample Collection and Plasma Protein Analysis

Venous blood samples were collected using a safety winged blood collection needle (0.6 mm × 19 mm × 305 mm) (Henry Schein; Melville, NY, USA) and 10 mL K2 EDTA coated vacutainers (Beckton Dickinson; Franklin Lakes, NJ, USA). Venous blood samples were taken prior to beginning the warm-up for a baseline measure and one hour post back-squat exercise stimulus. Collected blood samples were centrifuged at room temperature for 15 min at 3500 rpm (PowerSpin C856 Centrifuge; UNICO; Dayton, NJ, USA) for plasma separation. All plasma samples were stored at −80 °C until analysis. Venous blood samples were able to be collected on 10 of the 13 participants (1 female, 9 male) in the current study. The remaining 3 participants had incomplete venous blood samples and were excluded from analysis. Enzyme-linked immunosorbent assays were used to assess changes in plasma IL-6 (ab46042; Abcam; Cambridge, MA, USA), VEGF (ab222510; Abcam; Cambridge, MA, USA), and myoglobin (ELH-Myoglobin; RayBiotech; Peachtree Corners, GA, USA) according to manufacturer’s instructions. Following completion of assay procedures, changes in colorimetric absorbance were evaluated using a multi-mode plate reader (Synergy LX; BioTek Instruments; Winooski, VT, USA).

In addition to plasma protein analysis, finger-prick blood lactate samples were taken at multiple time points. Lactate measurements were taken prior to beginning the warm-up and twice following the back-squat exercise stimulus: immediately post exercise (before deflating the cuff in BFR condition), and 4 min post-exercise (2 min post cuff deflation in BFR). Capillary blood samples were drawn from the fingertip using contact-activated lancets (BD Microtainer; Beckton Dickinson; Franklin Lakes, NJ, USA) and were examined using a handheld lactate analyzer (Lactate Plus Meter; Nova Biomedical; Waltham, MA, USA).

#### 2.3.6. Performance and Perceived Exertion and Limb Pain

The number of back-squat repetitions completed during the exercise protocols was recorded for each set for both conditions. The total number of repetitions across all sets was determined from the sum of completed repetitions of four sets within each condition. Immediately after each set of back-squats, participants were asked about their perceived exertion and limb pain during the set. Ratings of Perceived Exertion (RPE) were assessed using a 0–10 scale (Borg CR-10), with 0 anchoring “nothing at all” and 10 being “impossible” [33]. Perceived limb pain was assessed using a standard numeric pain scale ranging from 0–10 (Borg CR-10) with 0 anchoring “no pain” and 10 being “extreme pain” [33]. Responses for RPE and Pain were recorded following each set on a physical data collection sheet.

#### 2.3.7. Maximal Voluntary Isometric Contraction

A strength assessment of the quadriceps and hamstring musculature was completed via MVIC of leg extension and flexion on the dominant leg. Leg dominance was determined by inquiring which side the participant would use to kick a ball at a target. The MVICs were performed using an isokinetic dynamometer (HUMAC NORM, CSMiSolutions, Stoughton, MA, USA). Participants were tested in a seated position at a 90° chair-back angle. A velcro strap was placed over the most distal portion of the thigh of the dominant leg performing the repetitions, directly superior to the knee joint to allow for greater stability and limit excessive movement of the leg during muscle contractions. The full range of motion (ROM) of the knee joint was first determined from a starting angle of 90° to full extension at 0°. Participants completed three repetitions of maximal isometric leg extension at a knee joint angle of 60°, with 0° being full extension [34]. Participants were instructed to kick out “as hard as possible” against the cushion and hold the contraction over the measurement period. Each MVIC was held for five seconds to attain peak torque (Nm), with one minute of rest given between repetitions. These procedures were conducted both pre and post exercise.

#### 2.3.8. Mean Propulsive Velocity

MPV was collected using a linear position transducer (GymAware PowerTool, Kinematic Performance Technology in Canberra, Australia). Participants performed three back-squats using 60% 1RM with a 1-min rest between each repetition. MPV (m/s) was averaged from the three repetitions. These procedures were performed both pre and post exercise.

#### 2.3.9. Countermovement Jump

Countermovement jump (CMJ) height was measured using portable force plates (Kistler 9286ba 10kn, Winterthur, Switzerland). Participants were instructed to stand at the center of the force plates and jump as high as possible while maintaining their hands on their hips. Participants performed three jumps with a 1-min rest between each jump. Flight height (m) was measured by Kistler MARS: Measurement, Analysis, and Reporting Software (Kistler, Kistler Instrument Group, Novi, MI, USA). Flight height was averaged from the three CMJs [35]. These procedures were conducted both pre and post exercise.

#### 2.3.10. Surface Electromyography

Pre-gelled disposable Ag/AgCl electrodes (EL503, Biopac Systems, Inc., Goleta, CA, USA) were placed on the dominant-leg rectus femoris (RF) and vastus lateralis (VL) in accordance with SENIAM recommendations. RF electrodes were placed midway between the anterior spine iliac and the top of the patella [36]. VL electrodes were placed 2/3 of the way between the anterior spine iliac and the lateral side of the patella [36]. The patella served as the reference electrode location. The electrode sites were shaved with a twin-blade single-use razor and cleaned/abraded with isopropyl alcohol prior to placing electrodes. Surface electromyography (sEMG) signals were sampled at 2 kHz with an electronic signal acquisition system (Biopac MP150 Physiograph, BIOPAC, Goletta, CA, USA), which was connected to a PC. sEMG signals were recorded for MVICs and MPVs pre and post exercise. EMG data were managed and analyzed using AcqKnowledge software (Version 4.4, BIOPAC, Goletta, CA, USA). The root mean squared (RMS) function was used for EMG signal analysis. The middle 500 ms epoch of the signal acquired during the five-second MVIC was analyzed. The middle 250 ms epoch of the signal acquired during the concentric portion of the MPV was analyzed.

### 2.4. Statistical Analyses

Data were managed using Microsoft Excel for Windows (Microsoft Corporation, Redmond, WA, USA). Statistical analyses were completed using SPSS for Windows (Version 25.0, IBM, Somers, NY, USA). All data are presented as mean ± standard deviation (M ± SD), unless otherwise noted. Mauchly’s test was used to test the assumption of sphericity and a Greenhouse–Geisser correction was applied when the assumption of sphericity was not met. All measured variables were analyzed by two-way repeated measures analysis of variance (ANOVA). Post hoc analyses following significant results from the ANOVAs were performed using *t*-tests and one-way ANOVAs, where appropriate. Bonferroni’s correction was used for adjustment for multiple comparisons. An alpha level of *p* < 0.05 was utilized to determine statistical significance. Partial eta squared (ηp^2^) were used to determine the magnitude of the effect size of main effects of condition and time and interaction effect (time × condition). Cohen’s *d* effect sizes were calculated to determine the magnitude of difference in measured parameters between conditions. Cohen’s *d* effect sizes values of 0.20, 0.50, and 0.80 corresponded to small, medium, and large effect sizes, respectively [37].

## 3. Results

### 3.1. Baseline Characteristics

Participant characteristics, including 1RM and LOP, are presented in Table 1. No significant differences were observed between visits in participant pre-performance characteristics, assessed by paired *t*-tests, VAS-DOMS (0.44 ± 0.54, 0.45 ± 0.58 arbitrary units (AU), respectively; *p* = 0.933), Perceptual Recovery Status (8.61 ± 1.12, 9.00 ± 0.82 AU respectively; *p* = 0.209), indicating similar participant pre-performance characteristics. Additionally, participants completed a self-paced warm-up (5 min cycle ergometer followed by a dynamic warm-up) which was repeated for the following visit. No significant difference existed in warm-up time between conditions (559.38 ± 130.65, 555.38 ± 146.66 s respectively; *p* = 0.790).

### 3.2. Repetitions

**Figure 1:**
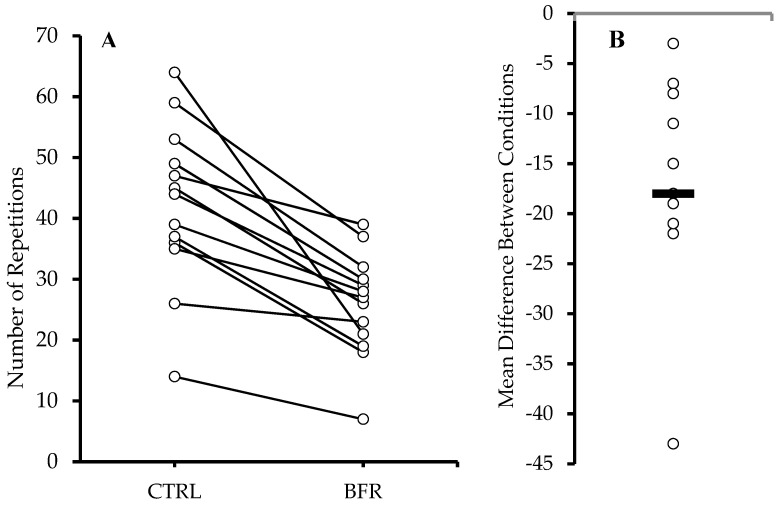
Total repetitions across all sets (**A**). Circles and connected lines represent individual responses. Mean difference between conditions (BFR−CTRL) (**B**). Circles and bar represent individual and group mean differences, respectively.

Significantly more total repetitions (Figure 1) were performed in the CTRL condition (42.15 ± 13.35 reps) compared to BFR (25.85 ± 8.48 reps) (*p* < 0.001). A significant time x condition interaction (F = 14.010, *p* < 0.001, ηp^2^ = 0.539), and main effects for condition (F = 34.040, *p* < 0.001, ηp^2^ = 0.739) and time (F = 41.670, *p* < 0.001, ηp^2^ = 0.776) were observed. Significantly greater number of repetitions were performed during the CTRL condition during sets 1, 2, and 4 (*p* < 0.05) compared to BFR, resulting in a significantly higher number of total repetitions during CTRL (Table 2).

### 3.3. Ratings of Perceived Exertion and Perceived Pain

A significant main effect for time was observed on RPE (F = 17.201, *p <* 0.001, ηp^2^ = 0.589), but no interaction (F = 2.320, *p* = 0.092, ηp^2^ = 0.162) or main effect of condition (F = 0.622, *p* = 0.445, ηp^2^ = 0.049). Both BFR and CTRL conditions demonstrated significant increases in RPE from set 1 to set 4 (*p* < 0.05) (Table 2).

Significant interaction (F = 4.624, *p* = 0.008, ηp^2^ = 0.278) and main effects for condition (F = 16.794, *p* = 0.001, ηp^2^ = 0.583) and time (F = 24.106, *p <* 0.001, ηp^2^ = 0.668) were observed in perceived pain. Perceived pain was significantly greater in the BFR condition across all sets (*p* < 0.05), when compared to CTRL (Table 2).

### 3.4. MVIC and Electromyography

There were no significant interaction effects for peak torque during isometric knee extension (F = 0.000, *p* = 0.986, ηp^2^ = 0.000) or isometric knee flexion (F = 0.105, *p* = 0.751, ηp^2^ = 0.009). A significant main effect for time (F = 12.253, *p* = 0.005, ηp^2^ = 0.527) was observed in isometric knee flexion, but no other significant main effects were observed in isometric knee extension or flexion peak torque. No significant interaction or main effects were observed for sEMG in the RF or VL during maximal isometric knee extensions (Table 3).

### 3.5. Countermovement Jump

A significant interaction (F = 5.527, *p* = 0.037, ηp^2^ = 0.315) and main effect for time (F = 87.327, *p* < 0.001, ηp^2^ = 0.897) on CMJ was observed. Significant reductions in CMJ height pre to post exercise were observed in the BFR (−12.27%, *p* < 0.001) and CTRL (−16.06%, *p* < 0.001) conditions. No significant difference in post-exercise CMJ height was observed between conditions (*p* = 0.093; Table 3).

### 3.6. MPV and Electromyography

A significant main effect for time on MPV was observed (F = 29.077, *p* < 0.001, ηp^2^ = 0.708), but no significant interaction (F = 0.410, *p* = 0.534, ηp^2^ = 0.033), or condition effect (F = 0.063, *p* = 0.850, ηp^2^ = 0.005) was observed. No significant interaction or main effect of condition were observed for MPV-sEMG in the RF or VL. Significant main effects of time were observed in the RF-sEMG (F = 4.982, *p* = 0.045, ηp^2^ = 0.293) and VL-sEMG (F= 9.493, *p* = 0.010, ηp^2^ = 0.442) during MPV testing, with significant decreases in MPV-sEMG observed in both muscles from pre to post exercise in the CTRL condition (Table 3).

### 3.7. Blood Lactate

**Figure 2:**
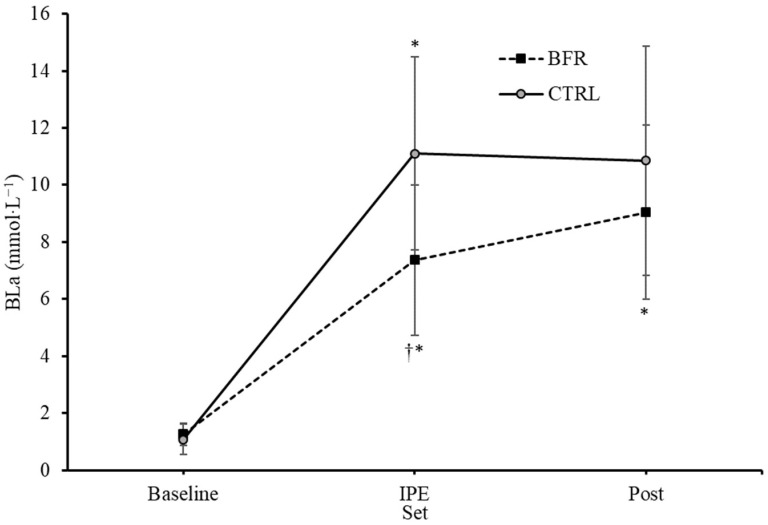
Changes in blood lactate concentrations (mmol·L^−1^) over time from Pre (baseline) to immediately post-exercise (IPE) and 4 min post (Post) exercise. BFR—blood flow restriction. CTRL—control. † Significant difference (*p* < 0.05) from CTRL. * Significant difference (*p* < 0.05) within condition compared to previous time point. Error bars represent standard deviation.

A significant interaction effect for BLa was observed (F = 15.126, *p* = 0.001, ηp^2^ = 0.558). Significant main effects on BLa for condition (F = 9.449, *p* = 0.010, ηp^2^ = 0.441) and time (F = 108.409, *p* < 0.001, ηp^2^ = 0.900) were observed. No significant differences in BLa were observed between BFR and CTRL at baseline (1.23 ± 0.40, 1.06 ± 0.51 mmol·L^−1^, respectively, *p* = 0.132) or four minutes post exercise (8.95 ± 2.95, 10.72 ± 3.90 mmol·L^−1^, respectively, *p* = 0.063) (Figure 2). BLa immediately post exercise was significantly greater in the CTRL condition (11.09 ± 3.24 mmol·L^−1^) compared to BFR (7.35 ± 2.53 mmol·L^−1^) (*p* = 0.001).

### 3.8. Interleukin 6

**Figure 3:**
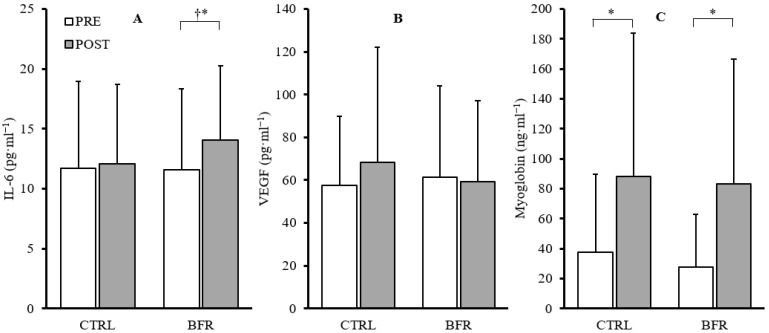
Changes in IL-6 (**A**), VEGF (**B**), and myoglobin (**C**) from pre (white) to post exercise (gray) in CTRL and BFR groups. † Significant difference (*p* < 0.05) from CTRL. * Significant difference (*p* < 0.05) within condition compared to PRE. Bars represent the mean and error bars represent standard deviation. Dots represent individual data points.

A significant main effect of time was observed in IL-6 (F = 10.803, *p* = 0.009, ηp^2^ = 0.546), but no significant interaction (F = 3.422, *p* = 0.097, ηp^2^ = 0.275) or condition effects (F = 1.285, *p* = 0.286, ηp^2^ = 0.125) were observed. Paired-samples *t*-tests within conditions indicated a significant increase in IL-6 in the BFR condition (+39.7%, *p* = 0.011) and non-significant increase in the CTRL condition (+11.11%, *p* = 0.595). Additionally, post-exercise IL-6 concentrations were significantly greater in the BFR condition (14.08 ± 6.18 pg·mL^−1^) compared to CTRL (12.06 ± 6.67 pg·mL^−1^; *p* = 0.007) (Figure 3).

### 3.9. Vascular Endothelial Growth Factor

No significant time*condition interaction effect (F = 2.023, *p* = 0.189, ηp^2^ = 0.184) or main effects for condition (F = 0.433, *p* = 0.527, ηp^2^ = 0.046) or time (F = 2.188, *p* = 0.173, ηp^2^ = 0.196) were observed in VEGF (Figure 3).

### 3.10. Myoglobin

No significant time*condition interaction effect (F = 0.066, *p* = 0.804, ηp^2^ = 0.007) or main effect of condition (F = 0.153, *p* = 0.705, ηp^2^ = 0.017) were observed. A significant main effect of time (F = 15.226, *p* = 0.004, ηp^2^ = 0.628) was observed. Significant increases in myoglobin were observed in both BFR (+201.73%, *p* = 0.007) and CTRL (+135.30%, *p* = 0.017) (Figure 3).

## 4. Discussion

To the best of our knowledge, this is the first study to examine the acute effects of bilateral limb BFR during high-intensity barbell back-squats. The purpose of this study was to examine the effect of bilateral BFR during high-intensity resistance exercise on performance and fatigue, metabolic stress, and plasma markers of muscle damage, inflammation, angiogenesis. Findings indicate the utility of bilateral HI-BFR back-squats for earlier onset of fatigue during exercise, with similar decrements in post-exercise performance, although associated with significantly higher pain perception. Additionally, the addition of BFR enhances acute post-exercise inflammation.

In this study, participants performed significantly fewer total repetitions in the BFR condition compared to the CTRL condition (25.8 ± 8.5 reps vs. 42.2 ± 13.4 reps, respectively). Contrary to our findings, Winchester et al. observed no difference in the total number of repetitions performed between BFR and CTRL (47.0 ± 4.25 vs. 44.92 ± 3.13, respectively; *p* = 0.29) during a barbell back-squat protocol (75% 1RM) until failure [20]. Unlike the current study, BFR was applied unilaterally and intermittently during exercise (pressure released during rest) [20].

Enhanced fatigue during BFR exercise has been associated with metabolic stress and the accumulation of metabolites. The restriction of blood flow and reduction in available O_2_ during BFR exercise increases the rate of PCr hydrolysis and P_i_ accumulation [14,38]. The increased metabolite accumulation can inhibit Ca^2+^ release and disrupt excitation-contraction coupling, resulting in peripheral muscular fatigue [39]. Additionally, the resynthesis of creatine phosphate during rest periods may be disrupted due to the limited O_2_ availability and H+ accumulation [40]. Contrary to our hypothesis, blood lactate was significantly greater in the CTRL condition immediately post exercise compared to BFR (11.09 ± 3.24, 7.35 ± 2.53 mmol·L^−1^, respectively); however, although CTRL remained elevated, no significant difference between conditions was observed 4 minutes post exercise (10.72 ± 3.90, 8.95 ± 2.95 mmol·L^−1^, respectively). While not matching the lactate levels of CTRL, BFR may have enhanced the blood lactate response while completing less total work during the exercise protocol. Teixeira et al. demonstrated that high-intensity resistance exercise with BFR applied only during the rest between sets (three sets of eight repetitions of unilateral knee extension) significantly increases blood lactate concentration compared to non-BFR or BFR during sets (un-occluded rest) [21]. In the present study, the removal of BFR during the rest period between sets 2 and 3 may have allowed for clearance of accumulated metabolites in the lower limbs. Average restricted time during the first inflation period (sets 1 and 2) was 341.9 (±24.0) seconds and 425.7 (±48.8) seconds for the second inflation period (sets 3 and 4). Total time with BFR applied during exercise was 767.6 (±64.9) seconds. The underlying reason for the release of BFR pressure during the rest period following the second set was the discomfort associated with BFR training, especially during high-intensity exercise [41].

Consistent with Winchester et al. [20], pain perception was elevated during the BFR condition across all sets in the current study. Pain perception during the CTRL condition was relatively constant across all sets (5.15–6.15), while the BFR condition experienced significantly elevated pain during set 2 (7.92) and set 4 (9.08). While anecdotal, participants had great difficulty managing pain during the occluded rest periods and this may have affected the ability to perform squat repetitions during the subsequent sets. While ratings of pain/discomfort have been found to not be associated with the relative pressure used or reductions in total repetitions at low intensity (30% 1RM) [42], the high mechanical load (75% 1RM) during this study may have exacerbated these effects. The un-occluded rest period following the second set was of great relief to participants and may, in part, explain the increased set performance during the third set.

Post-exercise fatigue was measured through changes in neuromuscular performance assessments and surface electromyography. Reductions in MVIC-EXT were non-significant, but consistent across conditions and MVIC-FLEX experienced significant reductions in peak torque which was also not different across conditions. Significant reductions, but consistent across conditions, were observed in MPV and CMJ, indicating neuromuscular fatigue [35]. Despite the exacerbated muscle fatigue during the BFR squat protocol, the effect sizes were larger in CTRL across all performance measures. This suggests that the impact of muscular fatigue during the exercise stressor was quickly reduced following the release of tourniquet pressure and reperfusion of the legs. These findings further validate the notion that reduced oxygen supply and accumulation of metabolites during BFR exercise results in peripheral muscular fatigue [39,43], and that reperfusion of the muscles post exercise quickly diminished this fatigue. In agreement with Neto et al. [23], decreases in MPV-sEMG were observed in both RF and VL following the exercise stimulus: BFR (−3.9% and −11.1%, respectively) and CTRL (−12.0% and −29.2%, respectively). Although non-significant, the reductions in sEMG were greater following the CTRL protocol and the effect sizes were larger. These differences may be related to the total mechanical load (total repetitions across four sets), leading to prolonged fatigue in response to the CTRL protocol. Therefore, while BFR experienced greater fatigue during sets, likely due to local metabolite accumulation, the total workload of the CTRL group potentially caused greater amounts of neuromuscular damage and longer-lasting fatigue.

Low-intensity (20% 1RM) BFR resistance exercise has been found to significantly increase plasma IL-6 following five sets of knee-extension to fatigue [44]. Winchester et al. found no significant differences between BFR (80% LOP) and traditional high-intensity back-squats (75% 1RM) in plasma IL-6 protein expression 1-hour post exercise (+31% and +22%, respectively, *p* < 0.05) [20]. Conversely, in the present study, a larger increase in plasma IL-6 was observed in the BFR condition compared to the CTRL condition (+39.7% vs. 11.11%, respectively). Winchester et al. [20] used unilateral intermittent limb BFR during exercise (pressure released during rest). The longer, more sustained intramuscular hypoxic environment through (segmented) continuous BFR protocol in the current study may be the underlying difference in IL-6 response. Other research has demonstrated that plasma IL-6 was elevated 2.6 fold after 45 min of aerobic exercise near anaerobic threshold [45]. These data lend support to the idea that tissue hypoxia and metabolic stress may be a determinant of IL-6 induction, which has been highlighted in past research [46].

In line with previous findings in low- [47] and high-intensity BFR exercise [20], observed changes in myoglobin concentrations were similar between the two conditions. These findings indicate that the addition of BFR during the high-intensity back-squat protocol in the current study did not result in enhanced muscle damage. However, the training induced muscle damaging effects of BFR exercise have been debated [48], and require further investigation at varying training intensities and volumes. Previous research in low-intensity BFR resistance exercise has shown significantly elevated plasma VEGF concentrations at 30, 60, and 120 minutes post exercise, and significantly greater concentrations 60 and 120 minutes post exercise compared a low-intensity control [49,50]. Takano et al. observed similar increases in plasma VEGF concentrations 10 and 30 minutes post LI-BFR resistance exercise in untrained adult men [51]. BFR during low-intensity resistance exercise and the associated reduced tissue oxygenation may promote VEGF secretion, as hypoxia is a stimulus of VEGF production [52]. However, no significant increases in VEGF were observed in the current study following HI-BFR. Interestingly, VEGF concentrations decreased following HI-BFR (−3.67%, 4.5 ± 4.5 pg·mL^−1^), while an increase was observed in CTRL (+18.70%, 26.83 ± 18.25 pg·mL^−1^) although neither post-exercise value was significantly different from pre-exercise (*p* < 0.05).

This study is not without limitations. Participants in the current study were classified as advanced resistance-trained and each had a minimum resistance training background of one year; therefore, the findings of the current study may not be applicable to untrained individuals or clinical populations. Total work (number of repetitions) was not matched between conditions and may have influenced the outcome in post-exercise measures, limiting interpretation. However, a goal of this study was to explore how BFR affects performance/fatigue during HI-RT, which traditionally has fewer repetitions per set than lower-intensity loads. A matched workload between conditions may allow for a better comparison of the acute effects between exercise conditions. Lastly, the BFR in the current study was interrupted following set 2, and therefore was not a truly continuous application of BFR during the exercise stimulus. As this was one of the first studies examining high-intensity resistance exercise in conjunction with BFR, the authors were cautious with the BFR application, and future research should examine truly continuous BFR with an optimized matched work–load exercise stimulus design. Additionally, future research may benefit from examining the utility of lower restriction pressures during HI-BFR to potentially decrease the perceived pain.

## 5. Conclusions

This study highlights that high-intensity BFR resistance exercise and non-occluded traditional high-intensity resistance exercise, performed to failure, cause similar post-exercise fatigue, blood lactate concentrations, and plasma myoglobin; however, BFR does so with 38.7% fewer repetitions. However, implementing high-intensity BFR in a resistance training protocol may prove difficult due to the significantly greater perceived pain throughout the workout. Therefore, this training method is not suited for rehabilitative purposes or use in untrained individuals. Rather, high-intensity BFR may prove useful for maximizing muscular hypertrophy and strength gains in bodybuilders and strength athletes aiming to accelerate their training progress. Future research should focus on the long-term safety, practicality, and efficacy of high-intensity BFR resistance exercise.

## Figures and Tables

**Table 1:** Descriptive characteristics of study participants.

	All (*n* = 13)	Female (*n* = 4)	Male (*n* = 9)
Age (yrs)	24.8 ± 4.7	23.8 ± 4.6	25.2 ± 4.9
Height (cm)	177.8 ± 11.8	164.1 ± 5.5	184.0 ± 7.6
Body Mass (kg)	84.3 ± 16.7	65.6 ± 3.6	92.5 ± 12.9
BMI (kg∙m^−2^)	26.4 ± 3.1	24.5 ± 2.0	27.3 ± 3.2
BF%	16.6 ± 8.2	24.0 ± 6.7	13.2 ± 6.6
Right LOP (mmHg)	219.6 ± 18.2	214.0 ± 10.7	222.1 ± 20.7
Right 80% LOP (mmHg)	175.6 ± 14.6	171.3 ± 8.6	177.6 ± 16.7
Left LOP (mmHg)	206.8 ± 25.8	204.8 ± 28.3	207.8 ± 21.1
Left 80% LOP (mmHg)	165.4 ± 20.7	163.8 ± 22.8	166.1 ± 21.1
1RM (kg)	143.7 ± 50.4	85.8 ± 15.2	169.4 ± 36.1

Values are mean ± SD. BMI—body mass index. cm—centimeters. BF%—body fat percentage. kg—kilograms. yrs—years. LOP—limb occlusion pressure. mmHg—millimeter of mercury. 1RM—one repetition maximum.

**Table 2:** Repetition, RPE, and Pain.

Variable	Condition	Set 1	Set 2	Set 3	Set 4
Repetitions	BFR	11.69 ± 4.19 ^†^	4.08 ± 2.30 ^†,^*	6.92 ± 3.16	3.15 ± 2.08 ^†,^*
	CTRL	13.54 ± 4.39	10.77 ± 3.49 *	9.15 ± 3.36 *	8.69 ± 3.60
Pain	BFR	6.58 ± 2.11 ^†^	8.00 ± 1.28 ^†,^*	7.33 ± 2.10 ^†^	9.17 ± 0.94 ^†,^*
	CTRL	5.25 ± 2.14	5.33 ± 2.15	5.75 ± 2.30	6.25 ± 2.53 *
RPE	BFR	8.46 ± 1.39	9.31 ± 0.85	9.15 ± 0.69 *	9.61 ± 0.51
	CTRL	8.54 ± 1.05	8.77 ± 0.83	9.23 ± 0.60	9.62 ± 0.60

Values are mean ± SD. BFR—blood flow restriction. CTRL—control. RPE—rating of perceived exertion. † Significant difference (*p* < 0.05) from CTRL; * significant difference (*p* < 0.05) within condition compared to previous set.

**Table 3:** Pre- and Post-Resistance Exercise Fatigue Measures.

Variable	Condition	Pre	Post	MD (95% CI)	Cohen’s *d*
MVIC Extension (Nm)	BFR	274.40 ± 81.81	267.25 ± 90.68	5.28 (−17.57, 28.14)	0.15
	CTRL	274.47 ± 89.95	267.02 ± 88.44	5.50 (−10.13, 21.14)	0.22
MVIC Flexion (Nm)	BFR	166.78 ± 58.89	155.24 ± 53.37 *	8.50 (0.44, 16.56)	0.67
	CTRL	163.65 ± 52.79	154.31 ± 54.39 *	6.89 (0.44, 13.33)	0.68
CMJ (m)	BFR	0.35 ± 0.07	0.31 ± 0.06 *	0.04 (0.03, 0.06)	2.25
	CTRL	0.35 ± 0.06	0.30 ± 0.05 *	0.06 (0.04, 0.07)	2.34
MPV (m·s^−1^)	BFR	0.71 ± 0.12	0.65 ± 0.14 *	0.07 (0.03, 0.10)	1.01
	CTRL	0.72 ± 0.10	0.64 ± 0.12 *	0.07 (0.05, 0.10)	1.84
MVIC sEMG RF (mV)	BFR	0.15 ± 0.09	0.17 ± 0.09	−0.11 (−0.03, 0.01)	−0.32
	CTRL	0.16 ± 0.07	0.19 ± 0.10	−0.03 (−0.07, 0.01)	−0.47
MVIC sEMG VL (mV)	BFR	0.17 ± 0.08	0.18 ± 0.09	−0.00 (−0.03, 0.01)	−0.09
	CTRL	0.19 ± 0.11	0.21 ± 0.14	−0.02 (−0.52, 0.00)	−0.57
MPV sEMG RF (mV)	BFR	0.26 ± 0.10	0.25 ± 0.10	0.01 (−0.25, 0.05)	0.19
	CTRL	0.25 ± 0.06	0.22 ± 0.06 *	0.03 (0.01, 0.04)	1.08
MPV sEMG VL (mV)	BFR	0.30 ± 0.13	0.27 ± 0.13	0.03 (−0.03, 0.10)	0.30
	CTRL	0.31 ± 0.10	0.24 ± 0.07 *	0.08 (−0.05, 0.10)	1.55

Values are mean ± SD. BFR—blood flow restriction. CTRL—control. MVIC—maximal voluntary isometric contraction. CMJ—countermovement jump. IHG—isometric handgrip. MPV—mean propulsive velocity. RMS—root mean square of the electromyographic signal. RF—rectus femoris. VL—vastus lateralis. MD—mean difference. Cohen’s *d*—Cohen’s *d* effect size. * Significant difference from pre within condition at *p* < 0.05.

## Data Availability

The data presented in this study are available upon request from the corresponding author.

## References

[1] Folland J.P., Williams A.G. (2007). The adaptations to strength training: Morphological and neurological contributions to increased strength. Sports Med..

[2] Cureton K.J., Collins M.A., Hill D.W., McElhannon F.M. (1988). Muscle hypertrophy in men and women. Med. Sci. Sports Exerc..

[3] American College of Sports Medicine (2009). Progression models in resistance training for healthy adults. Med. Sci. Sports. Exerc..

[4] Schoenfeld B.J. (2010). The Mechanisms of Muscle Hypertrophy and Their Application to Resistance Training. J. Strength Cond. Res..

[5] Sandri M. (2008). Signaling in muscle atrophy and hypertrophy. Physiology.

[6] Takarada Y., Takazawa H., Sato Y., Takebayashi S., Tanaka Y., Ishii N. (2000). Effects of resistance exercise combined with moderate vascular occlusion on muscular function in humans. J. Appl. Physiol..

[7] Takarada Y., Sato Y., Ishii N. (2002). Effects of resistance exercise combined with vascular occlusion on muscle function in athletes. Eur. J. Appl. Physiol..

[8] Vechin F.C., Libardi C.A., Conceição M.S., Damas F.R., Lixandrão M.E., Berton R.P., Tricoli V.A., Roschel H.A., Cavaglieri C.R., Chacon-Mikahil M.P. (2015). Comparisons between low-intensity resistance training with blood flow restriction and high-intensity resistance training on quadriceps muscle mass and strength in elderly. J. Strength Cond. Res..

[9] Lowery R.P., Joy J.M., Loenneke J.P., de Souza E.O., Machado M., Dudeck J.E., Wilson J.M. (2014). Practical blood flow restriction training increases muscle hypertrophy during a periodized resistance training programme. Clin. Physiol. Funct. Imaging.

[10] Winchester L.J., Morris C.E., Allen P., Wiczynski T., Arnett S.W., Lyons T.S. (2022). Effects of Varying Load Intensity on Skeletal Muscle Damage Between Two Isovolumic Resistance Exercise Bouts. Int. J. Exerc. Sci..

[11] Thiebaud R.S., Yasuda T., Loenneke J.P., Abe T. (2013). Effects of low-intensity concentric and eccentric exercise combined with blood flow restriction on indices of exercise-induced muscle damage. Interv. Med. Appl. Sci..

[12] Suga T., Okita K., Morita N., Yokota T., Hirabayashi K., Horiuchi M., Takada S., Takahashi T., Omokawa M., Kinugawa S. (2009). Intramuscular metabolism during low-intensity resistance exercise with blood flow restriction. J. Appl. Physiol..

[13] Pope Z.K., Willardson J.M., Schoenfeld B.J. (2013). Exercise and blood flow restriction. J. Strength Cond. Res..

[14] Suga T., Okita K., Takada S., Omokawa M., Kadoguchi T., Yokota T., Hirabayashi K., Takahashi M., Morita N., Horiuchi M. (2012). Effect of multiple set on intramuscular metabolic stress during low-intensity resistance exercise with blood flow restriction. Eur. J. Appl. Physiol..

[15] Moritani T., Sherman W.M., Shibata M., Matsumoto T., Shinohara M. (1992). Oxygen availability and motor unit activity in humans. Eur. J. Appl. Physiol. Occup. Physiol..

[16] Wernbom M., Jarrebring R., Andreasson M.A., Augustsson J. (2009). Acute effects of blood flow restriction on muscle activity and endurance during fatiguing dynamic knee extensions at low load. J. Strength Cond. Res..

[17] Yasuda T., Brechue W.F., Fujita T., Shirakawa J., Sato Y., Abe T. (2009). Muscle activation during low-intensity muscle contractions with restricted blood flow. J. Sports Sci..

[18] Doessing S., Heinemeier K.M., Holm L., Mackey A.L., Schjerling P., Rennie M., Smith K., Reitelseder S., Kappelgaard A.M., Rasmussen M.H. (2010). Growth hormone stimulates the collagen synthesis in human tendon and skeletal muscle without affecting myofibrillar protein synthesis. J. Physiol..

[19] Martineau L.C., Gardiner P.F. (2001). Insight into skeletal muscle mechanotransduction: MAPK activation is quantitatively related to tension. J. Appl. Physiol..

[20] Winchester L.J., Morris C.E., Badinger J., Wiczynski T.L., VanWye W.R. (2020). Blood Flow Restriction at High Resistance Loads Increases the Rate of Muscular Fatigue, but Does Not Increase Plasma Markers of Myotrauma or Inflammation. J. Strength Cond. Res..

[21] Teixeira E.L., Barroso R., Silva-Batista C., Laurentino G.C., Loenneke J.P., Roschel H., Ugrinowitsch C., Tricoli V. (2018). Blood flow restriction increases metabolic stress but decreases muscle activation during high-load resistance exercise. Muscle Nerve..

[22] Winchester L.J., Blake M.T., Fleming A.R., Aguiar E.J., Fedewa M.V., Esco M.R., Earley R.L. (2022). Hemodynamic Responses to Resistance Exercise with Blood Flow Restriction Using a Practical Method Versus a Traditional Cuff-Inflation System. Int. J. Environ. Res. Public Health.

[23] Neto G.R., Santos H.H., Sousa J.B., Júnior A.T., Araújo J.P., Aniceto R.R., Sousa M.S. (2014). Effects of high-intensity blood flow restriction exercise on muscle fatigue. J. Hum. Kinet..

[24] American College of Sports M., Riebe D., Ehrman J.K., Liguori G., Magal M. (2018). ACSM’s Guidelines for Exercise Testing and Prescription.

[25] Friedman K. (2016). Essentials of Strength Training and Conditioning, 4th Edition. Med. Sci. Sports Exerc..

[26] Beck T.W. (2013). The importance of a priori sample size estimation in strength and conditioning research. J. Strength Cond. Res..

[27] Whelton P.K., Carey R.M., Aronow W.S., Casey D.E., Collins K.J., Dennison Himmelfarb C., DePalma S.M., Gidding S., Jamerson K.A., Jones D.W. (2017). Guideline for the Prevention, Detection, Evaluation, and Management of High Blood Pressure in Adults. J. Am. Coll. Cardiol..

[28] Jackson A.S., Pollock M.L. (1985). Practical Assessment of Body Composition. Phys. Sportsmed..

[29] Siri W.E. (1956). The gross composition of the body. Adv. Biol. Med. Phys..

[30] Waterfield M.J., Sim J. (1996). Clinical assessment of pain by the visual analogue scale. Br. J. Ther. Rehabil..

[31] Laurent C.M., Green J.M., Bishop P.A., Sjökvist J., Schumacker R.E., Richardson M.T., Curtner-Smith M. (2011). A practical approach to monitoring recovery: Development of a perceived recovery status scale. J. Strength Cond. Res..

[32] Haff G.G., Triplett N.T. (2015). Essentials of Strength Training and Conditioning.

[33] Borg G. (1998). Borg’s Perceived Exertion and Pain Scales.

[34] Murray M.P., Gardner G.M., Mollinger L.A., Sepic S.B. (1980). Strength of isometric and isokinetic contractions: Knee muscles of men aged 20 to 86. Phys. Ther..

[35] Claudino J.G., Cronin J., Mezencio B., McMaster D.T., McGuigan M., Tricoli V., Amadio A.C., Serrao J.C. (2017). The countermovement jump to monitor neuromuscular status: A meta-analysis. J. Sci. Med. Sports.

[36] Hermens H.J., Commission des Communautés Européennes, Biomedical and Health Research Programme (1999). SENIAM: European recommendations for surface electromyography: Results of the SENIAM project. Roessingh Res. Dev..

[37] Cohen J. (1988). Statistical Power Analysis for the Behavioral Sciences.

[38] Sugaya M., Yasuda T., Suga T., Okita K., Abe T. (2011). Change in intramuscular inorganic phosphate during multiple sets of blood flow-restricted low-intensity exercise. Clin. Physiol. Funct. Imaging.

[39] Favero T.G., Zable A.C., Colter D., Abramson J.J. (1997). Lactate inhibits Ca(2+) -activated Ca(2+)-channel activity from skeletal muscle sarcoplasmic reticulum. J. Appl. Physiol..

[40] Sahlin K., Harris R.C., Hultman E. (1979). Resynthesis of creatine phosphate in human muscle after exercise in relation to intramuscular pH and availability of oxygen. Scand. J. Clin. Lab. Investig..

[41] Fitschen P.J., Kistler B.M., Jeong J.H., Chung H.R., Wu P.T., Walsh M.J., Wilund K.R. (2014). Perceptual effects and efficacy of intermittent or continuous blood flow restriction resistance training. Clin. Physiol. Funct. Imaging.

[42] Loenneke J.P., Kim D., Mouser J.G., Allen K.M., Thiebaud R.S., Abe T., Bemben M.G. (2016). Are there perceptual differences to varying levels of blood flow restriction?. Physiol. Behavior..

[43] Manini T.M., Clark B.C. (2009). Blood flow restricted exercise and skeletal muscle health. Exerc. Sport Sci. Rev..

[44] Takarada Y., Nakamura Y., Aruga S., Onda T., Miyazaki S., Ishii N. (2000). Rapid increase in plasma growth hormone after low-intensity resistance exercise with vascular occlusion. J. Appl. Physiol..

[45] Almada C., Cataldo L.R., Smalley S.V., Diaz E., Serrano A., Hodgson M.I., Santos J.L. (2013). Plasma levels of interleukin-6 and interleukin-18 after an acute physical exercise: Relation with post-exercise energy intake in twins. J. Physiol. Biochem..

[46] Pedersen B.K., Steensberg A., Fischer C., Keller C., Ostrowski K., Schjerling P. (2001). Exercise and cytokines with particular focus on muscle derived IL-6. Exerc. Immunol. Rev..

[47] Fujita T., Brechue W.F., Kurita K., Sato Y., Abe T. (2008). Increased muscle volume and strength following six days of low-intensity resistance training with restricted muscle blood flow. Int. J. KAATSU Train. Res..

[48] Wernbom M., Schoenfeld B.J., Paulsen G., Bjørnsen T., Cumming K.T., Aagaard P., Clark B.C., Raastad T. (2020). Commentary: Can Blood Flow Restricted Exercise Cause Muscle Damage? Commentary on Blood Flow Restriction Exercise: Considerations of Methodology, Application, and Safety. Front. Physiol..

[49] Patterson S.D., Leggate M., Nimmo M.A., Ferguson R.A. (2013). Circulating hormone and cytokine response to low-load resistance training with blood flow restriction in older men. Eur. J. Appl. Physiol..

[50] Jones M.T., Aguiar E.J., Winchester L.J. (2021). Proposed mechanisms of blood flow restriction exercise for the improvement of Type 1 diabetes pathologies. Diabetology.

[51] Takano H., Morita T., Iida H., Asada K., Kato M., Uno K., Hirose K., Matsumoto A., Takenaka K., Hirata Y. (2005). Hemodynamic and hormonal responses to a short-term low-intensity resistance exercise with the reduction of muscle blood flow. Eur. J. Appl. Physiol..

[52] Minchenko A., Bauer T., Salceda S., Caro J. (1994). Hypoxic stimulation of vascular endothelial growth factor expression in vitro and in vivo. Lab. Investig..

